# Propagation of Asian isolates of canine distemper virus (CDV) in hamster cell lines

**DOI:** 10.1186/1751-0147-51-38

**Published:** 2009-10-16

**Authors:** Serageldeen Sultan, Nguyen Thi Lan, Toshiki Ueda, Ryoji Yamaguchi, Ken Maeda, Kazushige Kai

**Affiliations:** 1 Department of Veterinary Microbiology, Faculty of Agriculture, Yamaguchi University, Yamaguchi 753-8515, Japan; 2 Department of Veterinary Pathology, Faculty of Agriculture, University of Miyazaki, Miyazaki 889-2192, Japan; 3 Department of Veterinary Pathology, Faculty of Veterinary Medicine, Hanoi University of Agriculture, Trau Quy-Gia Lam-Ha Noi, Vietnam

## Abstract

**Backgrounds:**

The aim of this study was to confirm the propagation of various canine distemper viruses (CDV) in hamster cell lines of HmLu and BHK, since only a little is known about the possibility of propagation of CDV in rodent cells irrespective of their epidemiological importance.

**Methods:**

The growth of CDV in hamster cell lines was monitored by titration using Vero.dogSLAMtag (Vero-DST) cells that had been proven to be susceptible to almost all field isolates of CDV, with the preparations of cell-free and cell-associated virus from the cultures infected with recent Asian isolates of CDV (13 strains) and by observing the development of cytopathic effect (CPE) in infected cultures of hamster cell lines.

**Results:**

Eleven of 13 strains grew in HmLu cells, and 12 of 13 strains grew in BHK cells with apparent CPE of cell fusion in the late stage of infection. Two strains and a strain of Asia 1 group could not grow in HmLu cells and BHK cells, respectively.

**Conclusion:**

The present study demonstrates at the first time that hamster cell lines can propagate the majority of Asian field isolates of CDV. The usage of two hamster cell lines suggested to be useful to characterize the field isolates biologically.

## Background

Canine distemper virus (CDV) is a negative-strand RNA virus that belongs to the genus *Morbillivirus *in the family *Paramyxoviridae*. The CDV induce distemper in dogs, raccoons [1] and ferrets [2]. Outbreaks of distemper in seals on Lake Baikal [3], in leopards and other feline animals in zoos [4], and in lions in the Serengeti National Park [5], indicate additional animal hosts other than the generally accepted hosts.

CDV has been isolated by a few cell lines other than the cells derived from dog as follows. Vero [6] and B95a B-cell line [7] were derived from monkey, and MV1Lu lung cells were derived from mink [8]. Recently, the gene of dog signaling lymphocyte activation molecule (also known as dog SLAM or CD150) [9], was introduced into Vero cells and Vero.dogSLAMtag (Vero-DST) cells were established [10]. By Vero-DST cells, various field strains were isolated [10-12]. Thus there are only a few cell lines derived from other than generally accepted hosts.

During a study on the molecular characterization of the hemagglutinin (H) and fusion (F) protein genes using mouse retrovirus vectors, the hamster HmLu cell line was suggested to be susceptible to CDV infection. Hamsters are not generally accepted hosts and there is little knowledge of the propagation of CDV in rodent cells.

## Methods

### Cells and Viruses

Vero-DST cells were established as described above [10]. HmLu cells were derived from hamster lung cells [13]. BHK cells were derived from baby hamster kidney cells [14]. All cells were passaged and maintained in Dulbecco's modified Eagle's medium (D-MEM; autoclavable; Nissui Pharmaceutical Co. Ltd., Tokyo, Japan) supplemented with 10% fetal bovine serum in a CO_2 _incubator at 37°C.

Thirteen strains of CDV were isolated and propagated, one or a few times, in Vero-DST cells and stored at -80°C until use. Specimens were collected from diseased dogs in Japan except for strain Th12, which was sampled in Thailand. Nine strains were newly isolated except for strains of 007 Lm, 009L, 011C [11], and Ac96I [12]. The phylogenetic relationship of nine new strains were determined by the sequencing of their (H) genes [15] where strains 55L, M25CR, 50Con and 50Cbl were assigned as members of Asia 2 group, while strains Th12, 50Sc, 81ND, 82Con and 83mLN were assigned as members of Asia 1 group. The vaccine strain of Onderstepoort (Ond) was used as a control.

### Virus titration

Vero-DST cells were seeded on 24-well multi-plates (Sumitomo-Bakelite, Tokyo, Japan) at a concentration of 1 × 10^5 ^cells per well and incubated overnight. After the removal of media, the cultures were overlaid with 0.1 ml of each serial 10-fold-diluted virus suspensions in duplicate manner, and incubated for 1 h with rocking at 15-min intervals. After virus adsorption, the suspensions were removed by suction and the cultures were washed once with culture media. The cultures were then overlaid with medium (1 ml) containing 1% methyl cellulose-4,000 (Nacalai Tesque, Kyoto, Japan; methyl cellulose media), incubated for 3 days, then fixed with formalin, stained with crystal violet solution, and plaques were counted. Virus titers were shown as mean value after two independent titrations which were carried out in a duplicate form.

### Passage of HmLu and BHK cells infected with various CDV strains

Sub-confluent cultures of HmLu or BHK cells were infected with strains Ond, 007Lm, 55L, 009L, M25CR, 011C, 50Con, 50Cbl, Ac96I, Th12, 50Sc, 81ND, 82Con, or 83mLN of CDV at a multiplicity of infection (MOI) = 1 in culture bottles. When the cultures became confluent (usually after 3-day incubation) the supernatant media was harvested, centrifuged at 3,000 rpm for 10 min to remove cell debris, aliquotted, and stored at -80°C until use as cell-free virus. The residual cells were liberated by trypsinization, and one tenth of the liberated cells underwent further cultivation, while the residual nine-tenths were used to liberate the cell-associated virus by freezing at -80°C, thawing under flowing water, and centrifugation at 3,000 rpm for 10 min to remove cell debris. The supernatant fraction was aliquotted and stored at -80°C until use as cell-associated virus. The cycle of passage was repeated, and all cell-free and cell-associated virus samples were titrated on Vero-DST cells.

## Results

### Propagation of CDV strains in HmLu and BHK cells

**Figure 1:**
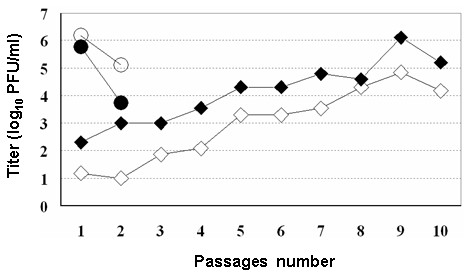
**Growth profiles of CDV strains of Ond and 007Lm in HmLu cells**. Viruses were harvested at every passage as cell-free viruses of Ondestepoort (open circle), or 007Lm (open diamond) and cell-associated viruses of Ondestepoort (black circle), or 007Lm (black diamond) and were titrated on Vero-DST cells as described in Materials and Methods. The obtained virus titers (PFU/ml) were plotted against passages number.

To find out whether the hamster cell lines can propagate CDV, a study was started with HmLu cells and CDV strains of Ond and 007Lm (Asia 2 group). When the HmLu cells were infected with the Ond strain at a MOI = 1, the virus induced the cytopathic effect (CPE) of giant cell formation, almost throughout the entire culture within 3-days. The obtained titers of cell-free and cell-associated viruses were 1.3 and 0.6 × 10^6 ^PFU/ml, respectively, at the 1^st ^passage. The passage of infected cells resulted in a low cell density and low virus titer, as shown in Fig. 1. The cell-associated virus titer obtained from HmLu cells infected with the 007Lm strain was 2 × 10^2 ^PFU/ml at the 1^st ^passage. The cell-free virus showed a titer that was about 10-fold lower (1.5 × 10^1 ^PFU/ml) than that of the cell-associated. The cell-free and cell-associated titers increased gradually up to the 9^th ^passage, and the highest virus titer (1.3 × 10^6 ^PFU/ml) was found for the cell-associated virus obtained at the 9^th ^passage. This score is comparable with that obtained for HmLu cells infected with the Ond strain. The persistent infection lasted during passages, although the CPE of giant cell formation appeared and increased gradually after the 4^th ^passage. The infected culture could be transferred up to the 15^th ^passage. On the other hand, the BHK cells infected with strain 007Lm were revealed to be transferred up to the 6^th ^passage (data not shown). According to these results, seven passages for infected HmLu and five passages for infected BHK cultures were preset for testing residual 12 CDV strains.

As shown in Table 1, all strains of Asia 2 group could grow in both HmLu and BHK cells. Among the strains of Asia 1 group, Ac96I could not grow in either HmLu or BHK cells and Th12 grew in BHK cells but not in HmLu cells. Moreover, strains 50Sc, 81ND, 82Con, and 83mLN could grow in either HmLu or BHK cells. The cultures of BHK cells infected with strains 50Cb1, Th12, or 81ND could not be transferred over 3 passages, the cultures of both HmLu and BHK cells infected with strain 82Con could not be transferred over 4 passages, and the cultures of HmLu cells infected with strains 55L, 009L, 50Sc, 81ND, and 83mLN also could not be transferred over 7 passages because the massive detachment of giant cells occurred, resulting in a low cell density with extensive appearance of CPE. A typical observation was that strain Th12 grew in BHK cells in a very short period of 3 passages but not in HmLu cells.

**Table 1:** The recovered virus titer from HmLu and BHK cells after several passages.

		**Titer (PFU/ml) recovered from**			
		
**Virus**	**Strain**	**HmLu (7P)^a)^**		**BHK (5P)^a)^**	
			
		**Cell-free**	**Cell-associated**	**Cell-free**	**Cell-associated**
	007Lm	3.4 × 10^3^	6.3 × 10^4^	6.0 × 10^5^	1.2 × 10^6^
	55L	2.5 × 10^5^	2.2 × 10^6^(7P)^b)^	1.6 × 10^5^	2.4 × 10^5^
	009L	1.0 × 10^5^	9.2 × 10^5^(7P)^b)^	3.0 × 10^5^	4.0 × 10^5^
Asia 2	M25CR	2.0 × 10^3^	8.0 × 10^5^	4.0 × 10^5^	2.0 × 10^5^
	011C	5.0 × 10^3^	2.0 × 10^4^	0.6 × 10^5^	1.6 × 10^5^
	50Con	6.0 ×10^3^	3.0 × 10^4^	2.5 × 10^5^	0.5 × 10^5^
	50Cb1	3.0 × 10^1^	2.2 × 10^2^	1.0 × 10^4^	7.0 × 10^3 ^(3P)^b)^

	Ac96I	< 5	≤ 5	< 5	< 5
	Th12	< 5	< 5	1.0 × 10^4^	8.0 × 10^3 ^(3P)^b)^
Asia 1	50Sc.	1.0 × 10^5^	1.9 × 10^5^(7P)^b)^	1.1 × 10^5^	1.9 × 10^5^
	81ND.	1.5 × 10^4^	2.5 × 10^4^(7P)^b)^	5.5 × 10^3^	1.5 × 10^4 ^(3P)^b)^
	82Con	1.2 × 10^6^	1.2 × 10^6^(4P)^b)^	4.5 × 10^5^	5.5 × 10^5^(4P)^b)^
	83mLN	2.0 × 10^5^	1.0 × 10^5^(7P)^b)^	1.3 × 10^5^	1.5 × 10^5^

^a) ^Preset passage level.

^b) ^Cultures at these passages were unable to be transferred further.

### Characterization of the CPE induced in hamster cells infected with CDV isolates

**Figure 2:**
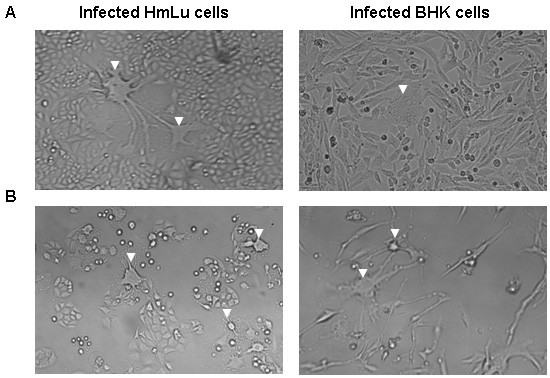
**The cytopathic effect (CPE) found in the cultures of HmLu cells and BHK cells infected with strain 007Lm**. The CPE were indicated by arrows. (A) Infected cultures at the middle stage of infection showing individual CPE among infected cells. (B) Infected cultures at the final stage showing destruction of cell layer and many CPE.

The culture of HmLu cells infected with strain 007Lm showed no CPE by the 3^rd ^passage as if strain 007Lm induced persistent infection. However, the culture showed CPE of giant cell formation (Fig. 2A) after the 4^th ^passage, and the population of giant cells increased gradually with increasing numbers of passages as the giant cells detached from the substrate as floating giant cells. This giant cell formation did not proceed to the formation of typical syncytia of fusion plaques that were observed in infection of Vero-DST cells with strain 007Lm by the 15^th ^passage although the population of giant cells increased. At the final stage, the culture showed extensive spread of giant cell formation and floating giant cells (round cells) throughout the entire culture (Fig. 2B). With regard to the infected BHK culture, similar process of CPE development was observed (Fig. 2A and 2B). To characterize giant cell formation, the culture of HmLu cells infected with strain 007Lm was liberated by trypsinization at the 8^th ^passage and plated into dishes to form colonies.

**Figure 3:**
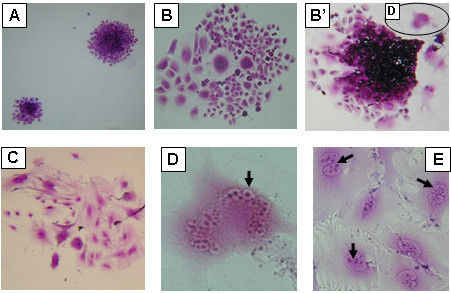
**Process of giant cell formation estimated by colony formation**. The morphology of colonies of HmLu cells infected with strain 007Lm at the 8^th ^passage. Colonies of normal cell shape (A). Hybrid colonies of normal and giant cells (B, B'). A colony composed of giant cells (C). A multinucleated giant cell had several individual nuclei, as indicated by arrow (D) magnified from D in (B'). Giant cells of big nuclei with several condensed materials as indicated by arrows (E). Magnification: 40× (A-C), and 200× (D and E).

The dishes were incubated for 7 days. Colonies of various sizes and morphologies were obtained and classified into three categories as shown in Fig. 3(A - C). Colonies with normal cell morphology, which represented the majority of colonies, as shown in Fig. 3A, hybrid colonies composed of normal and giant cell morphology are shown in Fig. 3B and 3B' (low incidence), and colonies composed of giant cells are shown in Fig. 3C (low incidence). Among the giant cells, a typical body, which seemed to be a nucleolus, was found as condensed material in the center area surrounded with a suggested nuclear membrane (Fig. 3D; indicated with an arrow). In other giant cells, several condensed materials were found in a circle (Fig. 3E; indicated with arrows).

Strains 55L, 009L, M25CR, 011C, 50Con, 50Cb1, 50Sc, 81ND, 82Con, and 83mLN induced similar CPE of giant cell formation in the infected HmLu culture at the middle and late stage of infection except at the first infection. Similar CPE of giant cell formation were also induced in the culture of BHK cells infected with every Asian isolate except for strain Ac96I.

## Discussion

BHK cells had a tendency to propagate field CDV strains more efficiently than HmLu cells as shown in the cases of strains 50Cbl, Th12 and 81ND (Table 1) and strain 007Lm. Fujita et al. [16] reported that BHK cells showed a low susceptibility to recombinant Yanaka virus but showed a high level CDV transcription. This high level CDV transcription might correlate to the rapid destruction of BHK cultures infected with strains 007Lm, 50Cbl, Th12 and 81ND when compared with the infected cultures of HmLu cells. However, strain 82Con grew in HmLu cells as rapidly as in BHK cells. Strain Ac96I could not grow in both HmLu and BHK cells and Th12 could grow in BHK cells but not in HmLu cells although it is not known what factor controls the growth of CDV in hamster cells at present. Thus, each virus strain behaved differently against the two hamster cell lines (Table 1). These results indicate that the usage of two hamster cell lines is useful to characterize various field strains by their biological nature.

In this report, all of Asian isolates induced no CPE at the initial stage of infection in the hamster cell lines of both HmLu and BHK, and this result is coincided with the reports [11,17] that both groups of Asia 1 and Asia 2 showed no CPE in Vero cells at the first infection. However, repeated passage of infected hamster cells resulted in the fact that even Asia 1 isolates except for Ac96I, could grow in hamster cell lines with apparent CPE of giant cell formation. Thus, it is necessary to characterize the infection to a cell line not only at the initial stage but also at the late stage of infection.

The HmLu cells infected with 007Lm had a high passage level of 15 passages, whereas the HmLu cells infected with Ond strain induced massive detachment of giant cells after the 1^st ^passage and were unable to be transferred further. These observations indicated that the development of CPE of giant cell formation in the 007Lm-infected HmLu cells was very slow. The slow development of CPE enabled the observation of various types of colonies at the 8^th ^passage as shown in Fig. 3. Giant cells seemed to be produced gradually from the infected cells with normal shape by cell fusion. Once giant cells were formed, they had a tendency to detach from the surface of the dish and turn into the floating giant cells.

## Conclusion

This report demonstrated at the first time that the majority of Asian field isolates tested were propagated in hamster cell lines of HmLu and BHK. Each virus strain behaved differently against the two cell lines. Since there is no effective method to characterize the field isolates of CDV biologically, the usage of two hamster cell lines can be useful.

## Competing interests

The authors declare that they have no competing interests.

## Authors' contributions

SS performed all experiments in this report under supervisory of KK and KM. N-TL and TU participated in isolation of field strains into Vero-DST cells from the samples of diseased dogs under supervisory of RY. All authors have read and approved the final manuscript.
